# Chemistry of Therapeutic Oligonucleotides That Drives Interactions with Biomolecules

**DOI:** 10.3390/pharmaceutics14122647

**Published:** 2022-11-29

**Authors:** Chisato Terada, Seiya Kawamoto, Asako Yamayoshi, Tsuyoshi Yamamoto

**Affiliations:** Graduate School of Biomedical Sciences, Nagasaki University, 1-14 Bunkyomachi, Nagasaki 852-8521, Japan

**Keywords:** oligonucleotide therapeutics, material symbiosis, oligonucleotides, phosphorothioate, antisense, siRNA

## Abstract

Oligonucleotide therapeutics that can modulate gene expression have been gradually developed for clinical applications over several decades. However, rapid advances have been made in recent years. Artificial nucleic acid technology has overcome many challenges, such as (1) poor target affinity and selectivity, (2) low in vivo stability, and (3) classical side effects, such as immune responses; thus, its application in a wide range of disorders has been extensively examined. However, even highly optimized oligonucleotides exhibit side effects, which limits the general use of this class of agents. In this review, we discuss the physicochemical characteristics that aid interactions between drugs and molecules that belong to living organisms. By systematically organizing the related data, we hope to explore avenues for symbiotic engineering of oligonucleotide therapeutics that will result in more effective and safer drugs.

## 1. Introduction

**Figure 1:**
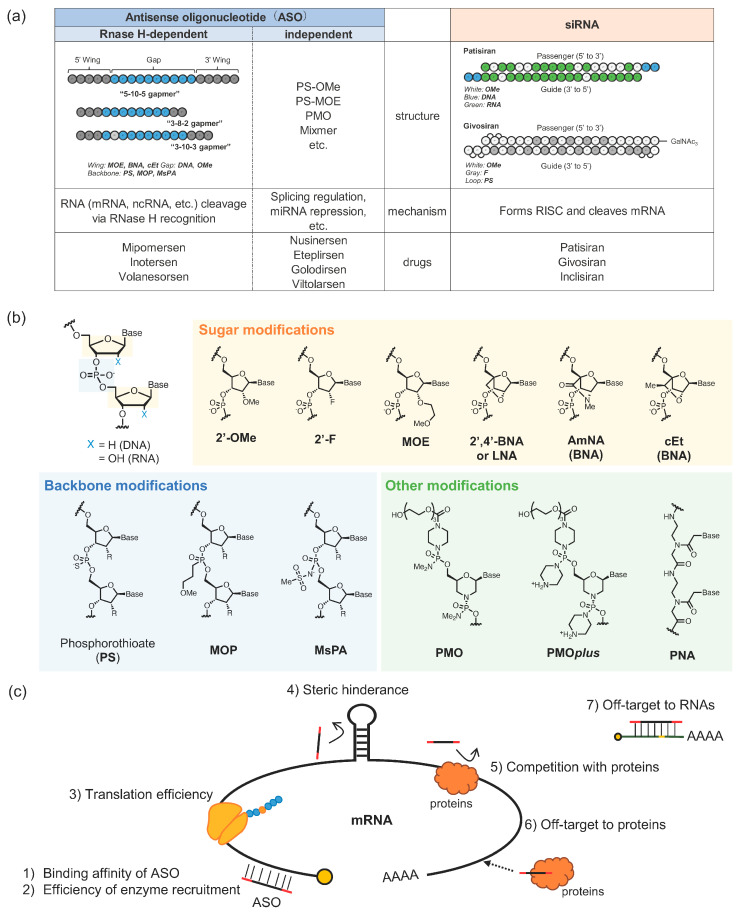
(**a**) Commonly used design and chemical modifications of antisense oligonucleotides (ASOs) and small interfering RNA (siRNA). (**b**) The chemical diversity of artificial nucleic acids used in therapeutic modalities. Chemical modifications are often categorized based on the component of the nucleotide (backbone, sugar, or base). (**c**) Factors affecting the functional expression of nucleic acids [2,3].

Nucleic acids were one of the most fundamental molecules generated on primitive Earth, and the evolution of molecular interactions between nucleic acids and their surrounding molecules represents the history of life. In recent years, “oligonucleotide therapeutics” have garnered interest as novel therapeutic modalities. Oligonucleotide therapeutics is a generic term for drugs with nucleic acids in their backbone. A wide variety of oligonucleotide-based therapeutics have been developed, including antisense oligonucleotides (ASOs), small interfering RNA (siRNA), aptamers, and decoys. Indeed, ASOs and siRNAs are synthetic nucleic acids designed to specifically modulate the expression, transcription, and translation of targets (Figure 1a). Superior backbone modifications have improved (1) affinity and selectivity to the target RNAs, (2) in vivo stability, and (3) the primary immune response (Figure 1b). However, owing to the “sticky” nature of the naturally-occurring nucleic acid molecules, highly optimized oligonucleotide drugs demonstrate a variety of side effects owing to some specific interactions with biological molecules and, hence, have not yet been adopted for widespread use (Spinraza^®^ is the only blockbuster among all the nucleic acid drugs that have been launched).

To reduce the side effects of oligonucleotide therapeutics and analyze their pharmacological effects, it is essential to foster a better understanding of their binding selectivity and specificity. Adapting the argument by Eaton et al. [1], the binding specificity ($\alpha_{s}$) of nucleic acids can be expressed in terms of thermodynamics, as in the following Equation (1):

(1) $$\alpha_{s}=\frac{K_{T}\left[T\right]}{\sum_{i}K_{O_{i}}\left[O_{i}\right]\;}$$

where *A* represents ASO, *T* represents the target RNA, *O_i_* represents the binding molecule other than the target RNA, $K_{T}$ specifies the binding constant between *A* and *T*, $K_{O_{i}}$ specifies the binding constant between *A* and *O_i_*, and [*T*] and [*O_i_*] represent the respective component concentrations. Binding specificity ($\alpha_{s})\;$has no direct relationship with binding constant with the target ($K_{T}$), indicating that balance with off-target binding reactions is important. Since a broad range of biological components bind nonspecifically to nucleic acids with affinities in the order of 10^−6^ M, it is assumed that oligonucleotides must have a dissociation constant of nM or less for adequately distinguishing between non-target and target molecules. Various interactions are observed in the cell, as shown in Figure 1c, and each could potentially affect the efficacy and safety of oligonucleotide therapeutics [2,3]. However, the fortification of off-target interactions of oligonucleotides by chemical modification has been key for successful improvements in the pharmacokinetics of oligonucleotide therapeutics. In this review, we classify and explain the interactions between nucleic acids (especially ASOs) and biomolecules by characterizing the process of the drug reaching target cells by interactions (1) in the blood, (2) on the cellular surface, and (3) inside the cell, to identify clues for achieving material symbiosis.

## 2. In-Plasma Dynamics of Oligonucleotide Drugs with Chemical Modifications

### 2.1. Oligonucleotide Therapeutics with Phosphorothioate Modification

Oligonucleotide therapeutics are now recognized as a reliable modality because of their improved thermodynamics attributed to chemical modifications. Oligonucleotide agents that enter systemic circulation from the site of administration are eliminated from the blood via various processes. Improvements in blood retention duration are important parameters that are directly related to availability in peripheral tissues. The half-life of natural oligonucleotides in blood is extremely short, ranging from 7.6 to 9 min [4]. The main route of elimination is degradation by nucleases; however, renal filtration (Mw < 40 kDa) also plays a significant role as the molecular weight of the agents is generally below the filtration limit (~5 kDa). Phosphorothioate (PS) modification, which replaces the non-bridging oxygen group with a sulfur atom, protects the phosphate backbone, which is a major target for nucleases, from hydrolysis. It increases the apparent molecular weight by improving interaction with the hydrophobic face of the protein, which is attributed to the hydrophobicity and polarization rate of sulfur. These characteristics associated with PS modification also allow oligo drugs to bypass renal filtration. This PS modification has been utilized for all ASOs and siRNAs, with a few exceptions (phosphorodiamidate morpholino oligomers [PMOs]). Therefore, PS modification is the de facto standard of systemically administered oligonucleotide therapeutics. At therapeutic doses, the blood protein binding rate of PS-modified oligonucleotide-based treatments is thought to be over 90%. Interactions between albumin, other blood proteins, and blood cell surface proteins have been confirmed [5]. The group led by Seth et al. at Ionis comprehensively and quantitatively analyzed the interaction of a gapmer-type PS-modified antisense nucleic acid containing 2′-*O*-methoxyethyl RNA (PS-MOEs) (Figure 1a), a sugar-modified RNA, with 25 major blood proteins using a fluorescence polarization (FP) assay [5]. The finding that the binding affinity of PS-MOE toward plasma proteins generally falls within the micromolar (10^−6^ M) range of the dissociation constants is particularly important. In human or mouse plasma, PS-MOEs primarily bind to albumin present in abundant amounts with a dissociation constant (*K*_d_) of approximately 12.7 µM and strongly bind to histidine-rich glycoprotein (HRG) with a *K*_d_ of 0.009 µM. Species differences may exist because the composition of plasma proteins varies among animal species. In addition, the interaction between PS-MOEs and proteins is strongly influenced by the number of PS linkages, chain length, number of charges, the isoelectric point of proteins, pH, and salt concentration. More impressively, when comparing the dissociation constants of various proteins with PS-modified dA 20-mer and dT 20-mer, the latter tend to show values that are smaller by two orders of magnitude, which indicates stronger binding [5,6]. Seth et al. concluded that the “flexibility” of oligonucleotides is also a pivotal factor in determining the interactions between nucleic acids and proteins because PS dA 20-mer shows stronger base-pair stacking interactions than PS dT 20-mer.

In contrast, side effects (such as a reduction in red blood cell counts, thrombocytopenia, prolonged activated partial thromboplastin time (aPTT), complement activation, and inflammatory reactions) observed in clinical trials can be attributed to the interaction between plasma components, such as blood cell components, clotting factors, and complement and nucleic acids, which are seemingly weak and minor [7,8,9]. While mipomersen is highly chemically modified to reduce lethal interactions, it shows a significantly prolonged elimination half-life in the blood (23–30 days), resulting in unintended long-term exposure in the blood [10]. Therefore, from the perspective of material symbiosis, it is necessary to scrutinize the introduction of new parameters, such as the duration of interactions and the unintended biological response elicited by such interactions (along with the biomarker search for quantitative evaluation), in addition to conventional parameters, such as dissociation constants and concentrations required for interactions.

Accumulating evidence in clinical trials has demonstrated the production of anti-drug antibodies (ADAs) against PS-MOEs [11,12]. After treating humans and monkeys with PS-MOEs, such as mipomersen, inotersen, and drisapersen, production of ADAs ranging from 20–70% was observed. The PS-MOEs comprise many diastereomeric mixtures as active ingredients because of the chirality of the phosphorous atom. Interestingly, ADAs are synthesized specifically from these active ingredients, even though the concentration of each isomer in the PS-MOEs is very low. This suggests that ADAs may not recognize the specific structure of nucleic acids precisely and robustly. However, such recognition may be mediated via some “weak interactions”. To the best of our knowledge, no report has demonstrated that production of ADAs reduces the effectiveness of oligonucleotide therapeutics. However, it is necessary to closely examine the mechanism of ADA production (e.g., T cell dependence), whether other chemical modifications of nucleic acids can also induce ADAs, and how ADAs recognize nucleic acids. In recent years, PS steric control has been examined [13,14,15], and the efficacy and safety of PS-modified nucleic acids with a single diastereomer have garnered significant interest [16,17]. Controlling PS conformation leads to minor differences in the orientation of the sulfur atom between the major groove and minor groove, affecting hydration, ion coordination, and the recognition of enzymes, such as RNase H. Progress in the research of nucleic acid chemistry is expected to enable more precise control of the interaction between nucleic acids and biomolecules.

### 2.2. Oligonucleotide Therapeutics with Phosphate Backbone Comprising Unnatural Modifications Other Than Phosphorothioate Modification

#### 2.2.1. Phosphorodiamidate Morpholino Oligomer (PMO)

Phosphorodiamidate morpholino oligomers have high nuclease resistance owing to the presence of electrically neutral and unnatural phosphate backbones called phosphorodiamidate linkages. However, the number of adverse events attributed to interactions with plasma proteins is lower than those observed with PS-MOEs because they generally do not interact with proteins as much as PS oligomers. However, PMOs tend to have lower tissue bioavailability and demonstrate effects at a higher dose (30–80 mg/kg/a week). The elimination half-life is approximately 2–15 h, which is shorter than that of PS-MOE [18,19]. Furthermore, their half-life in tissues is approximately 7–14 days. They are primarily excreted via urine in an unchanged form [20,21]. Three PMOs are commercially available, namely Vyondys53^®^, Viltepso^®^, and Amondys45^®^. These RNase H-independent ASOs act on the mRNA-encoding dystrophin protein in Duchenne muscular dystrophy and partially restore the function of the defective dystrophin protein by skipping specific exons. Analogs, such as PPMO with cationic peptides and PMOplus with partial positive charges developed by introducing piperazine residues, have been developed [22,23]. Although PPMOs with large cationic tails show dose-dependent toxicity (such as coma and weight loss) [24,25], a method of regulating the kinetics by utilizing unitized structures has the potential to fine-tune the physical properties and avoid class effects.

#### 2.2.2. Peptide Nucleic Acids (PNAs)

Nielsen et al. first reported a PNA with an electrically neutral aminoethyl glycine backbone in 1991 [26]. These PNAs are DNA mimics in which peptide-like backbones are substituted with negatively charged phosphodiester linkages. The PNA drug discovery has superior complementary DNA- or RNA-binding properties relative to its natural counterparts [27,28], and PNA-based drug discovery has uniquely enabled pharmaceutical development by targeting the RNA genome and oligonucleotide therapeutics. Because of its properties, PNA has been developed as a medicine; however, the desired therapeutic effect has not been observed because of the pharmacokinetics of PNAs [29]. The PNA only comprises four elements, namely hydrogen, oxygen, nitrogen, and carbon (HONC), and does not contain heavy atoms or other heteroatoms, such as fluorine. Elements relatively heavier than HONC, such as phosphorous, sulfur, and fluorine, have unique physicochemical and electronic properties and are essential for modulating molecular recognition in vivo. It must be noted that PNA differs from other nucleic acids comprising a phosphate backbone because it does not contain any of these elements and electrical charges.

In this Section, we summarize the interactions between the oligonucleotide class of drugs and plasma proteins. For nucleic acid therapeutics other than those involving PS-based modifications, data on the types of plasma proteins/components and the strength of their interactions is insufficient. Further research is necessary in the future.

## 3. Cellular Uptake of Oligonucleotides

### 3.1. Molecular Mechanisms of Cellular Intake

Oligonucleotide drugs interact weakly (*K*_d_~10^–6^ M) with carrier proteins in the systemic circulation, which enables the retention of compounds in the blood. It dissociates reversibly, and a free fraction can bind to the extracellular domain of the surface proteins on targeted cells. The fraction is subsequently absorbed and internalized. The PS-ASOs can interact (nonspecifically) with several types of membrane proteins, such as integrins, G protein-coupled receptors (GPCRs), receptor tyrosine kinases (RTKs), toll-like receptors (TLRs), epidermal growth factor receptors (EGFRs), scavenger receptor class B (SR-B), low-density lipoprotein receptors (LDLR), and asialoglycoprotein receptors (ASGPR) [30,31]. These receptors quickly retrieve PS-ASOs. It proceeds independently with energy, and the process can be saturated. The internalization process is affected by the type of membrane protein and the hardness of the lipid raft. Most of the aforementioned membrane receptors described are involved in clathrin-mediated endocytosis. Oligonucleotide drugs incorporated into this “productive” pathway efficiently translocate to the cytoplasm and rapidly localize to the nucleus and exhibit antisense activity. Integrin-mediated internalization and CLIC/GEEC pathways are also included in the production. In contrast, PS-ASOs consumed by high-capacity macropinocytosis tend to localize in lysosomes, which inhibits the antisense activity; hence, this process has been characterized as a “nonproductive” pathway.

Little is known about how PS-ASOs escape from endosomes after internalization via endocytosis. Nevertheless, some molecules have been identified that explain the trafficking of ASOs. For instance, ANXA2 localizes with PS-ASOs during endosome maturation. (They do not seem to interact directly with each other). In the absence of ANXA2, PS-ASOs remain in the primary endosome, resulting in a decrease in ASO activity. This explains why the protein may help promote drug escape to the cytosol when transported to late endosomes [32]. Similarly, GTPase RAB5C, a factor associated with the fusion of vesicular membranes, is essential for the uptake of PS-ASOs [33]. Lysophosphatidic acid (LBPA) could be an important player that helps ASOs escape from endosomes to the cytosol at a later stage [34]. Furthermore, LBPA is a phospholipid present in the inner membrane that is responsible for mass transport in and out of vesicles. A comparison of PS-ASO activity in various cancer cell lines showed that ASO knockdown tended to be lower in cell lines with a higher migration of PS-ASO to lysosomes, which is considered indirect evidence of escape into the cytoplasm during the early-late endosomal stage. This finding indirectly elucidates that during maturation, PS-ASOs escape into the cytosol from the primary endosome during subsequent stages [33].

Thus, it is assumed that the interaction with membrane surface proteins of the target cell (direct interaction between membrane lipids and therapeutic nucleic acids has not been reported) triggers the migration of oligonucleotides into endosomes via various endocytosis pathways and escape into the cytoplasm from late-stage endosomes. It is surprising that large negatively charged molecules, such as nucleic acids (although smaller than proteins), can escape directly into the cytoplasm, since large molecules, such as proteins, cannot escape from endosomes and are generally transported to lysosomes for degradation. In contrast, only approximately 0.1% of ASOs are able to enter the cytoplasm via these two processes [35].

### 3.2. Delivery of Nucleic Acid via Specific Molecular Interactions

Through the aforementioned nonspecific interactions between PS-ASO and proteins, it is clear that PS-ASO is distributed to and consumed by various tissues and organs throughout the body and exhibits knockdown activity [36]. In contrast, owing to concerns about their efficiency and side effects, targeting techniques have been developed to increase the selectivity of their distribution to target tissues [37]. In particular, we would like to focus on studies, including ours, on the ligand-targeted drug delivery (LTDD) method, which involves conjugation of a ligand for the engagement of therapeutic oligonucleotides with membrane proteins specifically expressed on the target cell surface.

#### 3.2.1. Asialoglycoprotein Receptor (ASGPR)

**Figure 2:**
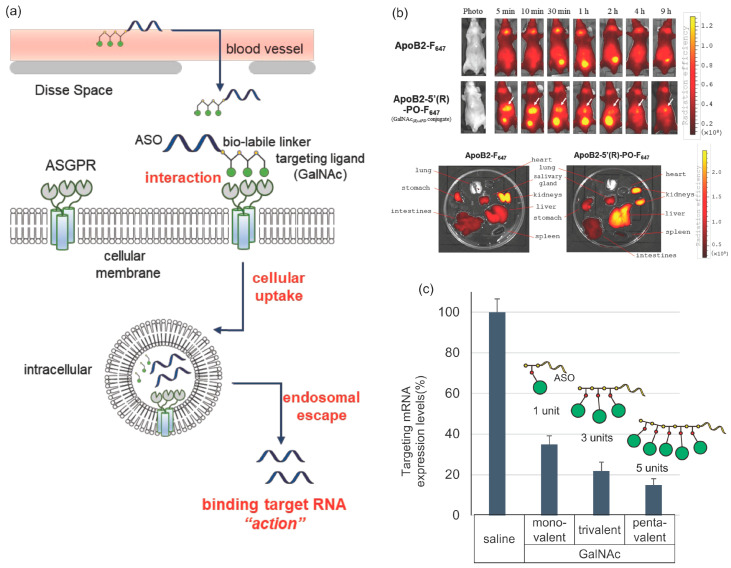
Distribution of *N*-acetyl galactosamine (GalNAc)-conjugated antisense oligonucleotide (ASO) and its mechanism of expression inhibitory activity. (**a**) Serial incorporation of monomeric GalNAc unit we developed previously to deliver ASOs to hepatocytes. Here, GalNAc is introduced into ASO via a biodegradable linker phosphodiester-linked monovalent GalNAc unit for effective delivery. (**b**) In vivo and ex vivo visualization of effect of monoanntenary GalNAc-conjugated ASO. Permission conveyed through Copyright Clearance Center, Inc. (**c**) Correlation between number of ligands and knockdown activity (modified based on references [45,46]).

The ASGPR lectin is one of the lectins discovered in the initial stage and is highly expressed in hepatocytes (0.7–5 × 10^5^ molecules per cell). Serum glycoproteins are promptly transported to the liver via receptor-mediated endocytosis. The major subunit H1 and minor subunit H2 form heterooligomers on the cellular membrane (Figure 2a) [38]. The receptor recognizes glycan proteins with *N*-acetyl galactosamine (GalNAc). Owing to the nature of GalNAc, its conjugation has been considered as a methodology for the hepatic drug delivery system (DDS) of therapeutic oligonucleotides.

The affinity of GalNAc for ASGPR is estimated to be very low (dissociation constant, *K*_d_~40 µM) [39]. Multivalency improves the binding affinity by the order of 10^−9^ M [40]. The first report on conjugates was published by Ts’o et al. in 1995 [41]. Since then, the activity of therapeutic oligonucleotides has been improving. In 2014, Prakash et al. showed that the binding of trimeric (triantennary-type) GalNAc ligands to ASOs with MOE or constrained ethyl bridged nucleic acid (cEt) can improve their knockdown activity in the liver by approximately 10-fold [42]. Similarly, a triantennary GalNAc-conjugated siRNA improved this effect [43]. The GIVLAAR^®^ and OXLUMO™ drugs, which have trivalent GalNAc, were the first approved drugs (siRNAs) worldwide.

In this context, we investigated the previously hypothesized trimeric ligand model [44]. Many previous studies have only evaluated the affinity of GalNAc with different valences for the ASGP receptor or its effect on cellular uptake, and the in vivo activity has not been adequately evaluated until recently. Hence, we developed a monomeric GalNAc phosphoramidite unit, with which we can freely change the ligand valency, and introduced it into PS-ASO equipped with bridged nucleic acids (2′,4′-BNA/LNA). Unexpectedly, a remarkable improvement in knockdown activity was observed with the introduction of only one GalNAc (Figure 2b,c) [45,46,47]. This suggests that the univalent GalNAc targets are clustered with ASGPR. This weak interaction may be favorable for efficient turnover of the ASO influx cycle into the liver.

#### 3.2.2. Glucagon-Like Peptide-1 Receptor (GLP1R)

The GLP1R is a GPCR that belongs to the secretin receptor family and is highly expressed, especially in the pancreas. In the pancreatic islets of Langerhans, they are present in insulin-secreting cells and somatostatin-secreting β cells. When GLP1R is activated, it is rapidly consumed by the cell and subsequently recycled. Ämmälä et al. have developed a GLP1R peptide agonist (eGLP1R) that fuses human GLP1 with a GLP1-like peptide fragment named exenatide [48,49]. The GLP1R peptide agonist (eGLP1), which is a fusion of human GLP1 and exenatide, was conjugated to ASO via biodegradable phosphate diester and disulfide bonds [48,49]. Typically, the binding of GLP1R with the GLP1 peptide is very strong (*K*_d_~0.5 nM), and this eGLP1 conjugate also demonstrates a potent activity with the GLP1 receptor. The eGLP1-mediated ASO showed no activity in other organs, including the liver, and showed pancreatic-cell-selective target gene knockdown. The eGLP1 pathway is considered to be a productive pathway. This pioneering study demonstrated delivery outside the liver using a ligand conjugation method. The potential impact of the remaining agonist activity on the ligand in the system should be examined in future studies.

#### 3.2.3. Glucose Transporter (GLUT1)

Miyata et al. focused on the glucose transporter GLUT1, which is highly expressed in the brain, for the delivery of therapeutic oligonucleotides to the brain [50]. The GLUT1 transporter is abundant in the endothelium of brain capillaries and migrates from the apical to the basal side in response to blood glucose. Based on this principle, they modified the surface of nanomicelles with glucose at various densities via the hydroxyl group present at the sixth position. At an optimal density, the encapsulated PS-ASO could be delivered into the brain parenchymal cells very efficiently and showed high knockdown activity. At this time, approximately 6% of the administered nanomicelles migrated into the brain. Notably, the dissociation constant of GLUT1/Glucose at 3 mM was very high. Again, by utilizing the multivalency effect, a weak interaction is converted into a strong one while ensuring specificity, and high levels of activity were successfully observed.

#### 3.2.4. Specific Delivery Using Different Kinds of Cell Surface Receptors

Exosomes are extracellular vesicles with a diameter of approximately 100 nm that contain a variety of proteins and nucleic acid molecules and serve as carriers for transporting these biomolecules between cells. Various exosomal surface antigens are known, including tetraspanin proteins (CD63, CD9, and CD81), cell adhesion molecules (integrin and ICAM-1), and HLA-G, an MHC class I molecule. The intracellular uptake of exosomes released into the blood is cell-directed according to the expression patterns of these antigens. Because of these characteristics, they have garnered interest as nanocarriers that can encapsulate drugs, such as liposomes, and deliver them organ-selectively. The most distinctive features of ExomiR-Tracker [51], a DDS technology we have developed, are listed as follows: (1) there is no need to isolate and purify exosomes, and (2) there is no need to encapsulate therapeutic nucleic acids inside the exosomes. Specifically, ExomiR-Tracker is a nucleic acid–antibody complex conjugated with an anti-Exo antibody that binds to exosome membrane surface proteins and anti-miRNA-ASO. We successfully delivered this complex into the bloodstream to capture target exosomes floating in the blood and effectively delivered therapeutic oligonucleotides to targeted cancer tissues. This study demonstrates that it is possible to achieve high target selectivity and to provide the higher level of functionality necessary to achieve material symbiosis by successfully hijacking a superior intrinsic system, such as exosomes, by nestling in and hiding from it, similar to a clown shark.

## 4. Intracellular Kinetics of Nucleic Acid Drugs and Their Regulation

An interesting study of the intracellular dynamics of PS-ASO was reported by Mundigle et al. [52]. When PS-ASO was introduced directly into the cytoplasm of MCF-7 cells by microinjection, most PS-ASO accumulated in the nucleus within five minutes (no migration into the nucleolus was observed). Since depletion of cellular ATP had no effect on this rate, it was concluded that nuclear migration is energy-independent and can be attributed to passive transport. The PS-ASO enter the nucleus during their free movement through the cytoplasm, where they are shackled. In this Section, we will examine the interactions between nucleic acids and substances in the cell that have a major impact on the drug efficacy, toxicity, and kinetics of therapeutic nucleic acids.

### 4.1. Interactions between Nucleic Acids and Organelles

**Figure 3:**
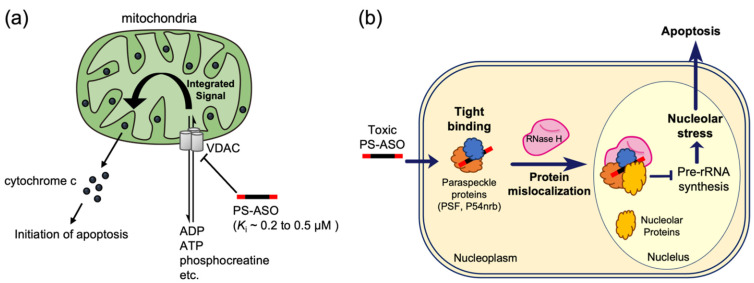
(**a**) A Hypothetical model in which PS-ASO inhibits VDAC and induces apoptosis. The interaction of PS-ASO with VDAC causes VDAC closure, which facilitates the release of cytochrome c through some not clearly identified mechanism (modified based on references [53,54]). (**b**) Toxic mechanisms of PS-ASOs mediated by interactions with paraspeckle proteins. Toxic PS-ASOs tightly bind intercellular proteins. The tight interactions can cause paraspeckle protein mislocalization to the nucleolus in a RNase H1-dependent manner, and can infect pre-rRNA synthesis, causing nucleolar stress and inducing apoptosis (modified based on reference [55]).

**Mitochondria**—Stein et al. reported that PS-ASO may interact with mitochondria from the outer membrane side (*K*_i_~0.2 to 0.5 µM) with mitochondrial voltage-dependent anion channels (VDAC) and inhibits mitochondrial respiration. This may trigger the release of cytochrome c and induce apoptosis (Figure 3a) [53,54].

**Nucleolus**—The function of toxic PS-ASOs is inhibited via interaction with the paraspeckle protein P54nrb in the nucleolus (Figure 3b) [55,56]. Surprisingly, replacing a portion of the gap region created from the central DNA of the toxic PS-cEt ASO with 2′-OMe RNA reduces its interaction with the P54nrb protein and prevents toxic effects. Moreover, the effect of this 2′-OMe RNA introduction appears to be adaptable to PS-cEt ASOs with various toxicities, which seems to be the main cause of tissue damage induced by these RNase H-dependent ASOs. Independent corroborative studies, examining the generality of the effect of the modification of this gap region, would be interesting. In cells, many droplet-like organelles are composed of these RNA-protein complexes due to phase separation phenomena, and it is thought that therapeutic nucleic acids interfere with their functions. We will continue to monitor the effects of other chemical classes of therapeutic nucleic acids on the function of these organelles.

### 4.2. Interactions with Proteins

The PS-ASO interacts with a variety of functional proteins in the cytoplasm and blood. In particular, Crooke et al. at Ionis have conducted quantitative and detailed analyses of the interaction of PS-ASO with proteins for many years (Table 1) [31]. From HeLa cell lysates, 58 proteins that bind to PS-ASO were identified by MS/MS analysis [57,58]. The proteins identified in this study were mostly known nucleic acid-binding and chaperone proteins. Crooke et al. performed thorough knockdown experiments to identify those that affect antisense activity and kinetics. Among these, HSP90 was suspected to be involved in this mechanism as its antisense effect was attenuated by knockdown. Detailed analysis revealed that PS-ASO binds to and enhances the activity of artificial nucleic acids with high hydrophobicity on the 5′ side. Other factors, namely Ku70, Ku80, P54nrb, and hnRNPs, were found to act competitively with RNase H1 and inhibit its antisense activity. Furthermore, they developed a unique interaction analysis system based on the nanoBRET system to effectively evaluate the stoichiometry ratio and dissociation constant of the binding between PS-ASO and target proteins [59]. The nanoBRET system uses the interaction between a luciferase fusion protein called NanoLuc and fluorescently labeled PS-ASO. This analysis revealed that the major PS-ASO-binding proteins described above have a binding strength of approximately 10^−9^ M. In addition to the fact that large differences can be observed in dissociation constants due to differences in sugar moiety modifications, significant results providing quantitative information on binding were obtained, including the possibility that *K*_d_ can vary by approximately 1000-fold for the same PS-cEt chemistry depending on the sequence.

Crooke et al. also actively investigated the impact of these factors on toxicity, focusing on whether colocalization was observed in the cells [55,60]. They also examined the impact of knockdown. For example, they believed that DDX21, P54nrb, and PSF [55] may contribute to toxicity.

Ionis et al. were the first to show that many of these intracellularly bound proteins show strong interactions at approximately 10^−9^ M [59]. It is not surprising that some of these proteins act as switches that turn on signals for toxicity. According to Crooke et al., the drawback of this proteomic analysis system is that only proteins that are relatively abundant and tightly bound to the cell can be observed [59]. In addition to constructing a system that can evaluate the binding of even small amounts of proteins, further studies are needed to develop a method for the analysis of proteins that play important roles through weak interactions with therapeutic oligonucleotides.

### 4.3. Interactions with Intracellular Nucleic Acids

The ASOs and siRNAs act on RNA. The stronger the binding of the drug with the receptor, the more potent is the expected pharmacological effect. From this perspective, the discovery of bridged nucleic acids has made it possible to remarkably increase the binding affinity with target RNAs by incorporating BNAs onto PS-ASO, thereby achieving clear in vivo drug efficacy enhancement [61]. Many BNA analogs have been developed and are widely used in medicine. However, strong tissue damage was observed in scattered cases of PS-ASO, especially with analogs of BNA, leading to the hypothesis that the toxicity may be caused by hybridization-dependent off-target knockdown, where ASO binds to non-target RNA and mediates expression suppression. This is based on the fact that there is a correlation between the ability to bind to target RNA and the frequency of the observation of toxic effects [62], where toxicity decreases when RNase H1 is deleted (unintended RNA cleavage no longer occurs) [63], and the higher number of complementary target sequences is correlated with higher toxicity [64].

## 5. Conclusions

Our goal is to generalize and describe the physicochemical properties required of therapeutic oligonucleotides in order to realize the “material symbiosis” between living organisms and oligonucleotide drugs. From this perspective, this review provides an overview of the various interactions of oligonucleotides in vivo. We provide a glimpse of the improvements made in the efficacy, pharmacokinetics, and safety of therapeutic oligonucleotides through efforts to quantitatively capture the molecular interactions of oligonucleotide drugs without compromising their therapeutic effects. In particular, for material symbiosis with therapeutic nucleic acids, it seems necessary to pay attention to the “quality” of binding with non-target molecules, in addition to the concept of binding specificity mentioned at the beginning of this article. This issue of safety hinders their practical application. Whether toxicity is hybridization-dependent or independent is hotly debated, and both theories are persuasive. Further experimental support or breakthrough technologies need to be developed in the future.

## Figures and Tables

**Table 1:** Proteins which interact with nucleic acid drugs and its dissociation constant (modified based on references [5,31,59].

Plasma Protein	*K*_d_ (10^−6^ M) (FP Assay)	MW (kDa)	Plasma Concentration (10^−6^ M)
Albumin	12.7	69	600
Immunogloblin gamma-1 heavy chain (IgG)	1.6	150	75
Apolipoprotein A-I (ApoA-I)	5.3	30	40
Apolipoprotein A-II (ApoA-II)	>500	11	24
Complement Factor C3	0.5	187	20
Transferrin	7	77	12
Alpha-1-Antitrypsin	>100	46	11
Haptoglobin	54.7	45	11
Hemopexin	13.9	52	9.9
Fibrinogen	0.87	24	9
Alpha-2-macroglobulin (A2M)	0.05	163	6
Transthyretin (TTR)	132	16	6
Antithrombin III (ATIII)	8.7	52	3.5
Alpha-1-antichymotrypsin (ACT)	21.3	47	3.3
Beta-2-Glycoprotein	57.1	38	2.7
Ceruloplasmin	22.6	122	2
Alpha-1-Acid Glycoprotein	>500	23	1.7
Complement Component C1q	3.4	26	1.6
Complement Factor C4	0.43	192	1.4
Histidine–rich glycoprotein	0.009	59	1.3
Plasminogen	2.1	90	1.2
Fibronectin (FN)	0.54	272	0.9
Apolipoprotein B-100 (Apo B-100)	>10	515	0.7
Factor H	0.5	139	0.6
Apolipoprotein E (Apo-E)	0.027	36	0.5
Factor V	0.032	251	0.02
**Intracellular protein**	***K*_d_ (10** **^−6^ M) (BRET assay)**		**MW (kDa)**
	**cEt**	**MOE**	
Leucine-rich PPR motif-containing protein, mitochondrial (LRPPRC)	0.16	0.77	41
RNA-binding protein FUS (FUS)	0.6	1.8	52
Proprotein convertase subtilisin/kexin type 4 (PC4)	1.1	6.1	14
60S ribosomal protein L5 (RPL5)	1.3	3.7	20
Nucleolin (NCL [RBD 1-4])	1.7	0.009	39
Splicing factor, proline- and glutamine-rich (SFPQ)	2.7	3.7	76
X-ray repair cross-complementing protein 6 (Ku70)	4	15	69
Ribonulease H1 (RNase H1)	5	2	32
Lupus La protein (La)	5	9.3	46
Non-POU domain-containing octamer-binding protein (NonO protein, P54nrb)	9.3	82.9	54
60S ribosomal protein L11 (RPL11)	17.4	15.7	34
Heat shock protein 90 (HSP90 [mid])	43	167	47
Staufen	100	-	55
T-complex protein 1 subunit beta (TCP1-B)	113	398	57
Actin, cytoplasmic 1 (ACTB)	295	252	42
Nuclear matrix protein 1 (NMP1)	>1000	>1000	28
Annexin A2 (ANXA2)	>1000	>1000	38

## Data Availability

Not applicable.
